# The use of automation in the rendition of certain articles of the Saudi Commercial Law into English: a post-editing-based comparison of five machine translation systems

**DOI:** 10.3389/frai.2023.1282020

**Published:** 2024-01-12

**Authors:** Rafat Y. Alwazna

**Affiliations:** Department of Modern Languages and Literature, Faculty of Arts and Humanities, King Abdulaziz University, Jeddah, Saudi Arabia

**Keywords:** machine translation, Arabic–English legal translation, post-editing, HTER metric, comparison, output quality, system

## Abstract

Efforts to automate translation were made in the 1950s and 1960s, albeit with limited resources compared to current advanced standards. Machine translation is categorised under computational linguistics that examines employing computer software in the rendition of text from one language into another. The present paper seeks to compare five different machine translation systems for the sake of assessing the quality of their outputs in rendering certain articles of the Saudi Commercial Law into English through post-editing based on Human Translation Edit Rate. Each machine translation output is assessed against the same post-edited version, and the closest output to the post-edited version with regard to the use of the same lexicon and word order will achieve the lowest score. The lower the score of the machine translation output is, the higher quality it has. The paper then analyses the results of the Human Translation Edit Rate metric evaluation to ascertain as to whether or not high-quality machine translation outputs always produce acceptable Arabic–English legal translation. The present paper argues that the use of Human Translation Edit Rate metric is a useful tool for the sake of undertaking post-editing procedures as it is a combination of both human evaluation as well as automatic evaluation. It is also advantageous as it takes account of both the use of lexicon and word order. However, such metric cannot be sufficiently depended on as one term substitution, which will be counted according to this metric as a single error, may render the whole sentence invalid, particularly in legal translation. This paper offers a baseline for the quality assessment of machine translation output through post-editing based on Human Translation Edit Rate metric and how its results should be analysed within Arabic–English legal translation context, which may have implications for similar machine translation output quality assessment contexts.

## 1 Introduction

Machine translation (MT) is deemed amongst the first non-numerical applications of the digital computers which appeared in the aftermath of World War 2 (Kenny, 2022). Efforts to automate translation were made in the 1950s and 1960s, albeit with limited resources compared to current advanced standards (Hutchins, 2000). MT is categorised under computational linguistics that examines employing computer software in the rendition of text from one language into another (Costa-Jussà et al., 2012). MT includes the electronic production of a receptor text based on an original text (Kenny, 2022). The complexity of natural languages makes MT an arduous task. Such complexity appears more clearly in the multiplicity of meanings of words, the different interpretations of sentences and the existence of certain grammatical relations in a particular language, but not in the other (Costa-Jussà et al., 2012). With this in mind, MT systems make a diverse set of errors, such as lexical, grammatical, syntactical, collocational and stylistical errors alongside unnecessary insertions or deletions (O'Brien, 2022).

Much research has been devoted to addressing MT systems translation quality, assessing their translation outputs, exploring ways of error correction through post-editing MT systems outputs and contributing to the improvement of their outputs through pre-editing the original texts to make them more translatable (Kenny, 2022). However, the present paper is distinctive as it compares five different MT systems' outputs that can work with Arabic texts, namely: Google Translate, PROMT.One, SYSTRAN Translate, Microsoft Bing and Translate.com in rendering two legal articles, namely: Article 17 and Article 108 from the Saudi Commercial Law into English. This runs in line with the evidently witnessed development in the area of the legal informatics that has been made in the last couple of years (Sharma et al., 2021). The comparison is based on post-editing rather than automatic evaluation as the latter involves a higher error rate as concluded by Koehn (2020) as well as Rossi and Carré (2022). This is owing to the fact that the latter is based on the comparison between the MT system output and a completely independent human translation that has nothing to do with the MT system output, whilst the former is grounded in the comparison between the MT system output and a post-edited version of that MT system output. The paper also analyses the results of the post-editing procedures based on Human Translation edit rate (HTER) to test as to whether or not such metric can be depended on in MT output assessment of Arabic–English legal translation. The significance of the current research lies chiefly in the notion that it uses automatic metric to carry out human post-editing then performs analysis of the results reached by post-editing based on HTER to test as to whether or not the five MT outputs in question are acceptable. The paper seeks to present how MT systems usually work with Arabic–English legal translation, particularly when they are given short legal articles and how they are assessed.

The present paper seeks to answer the following research questions: (1) What is the highest output quality of the five MT systems involved in the current study in the translation of certain articles of the Saudi Commercial Law into English according to HTER metric? (2) Do always the MT systems that have high outputs quality according to HTER metric produce acceptable Arabic–English legal translation, and why?

The present paper starts at the outset by providing a relatively succinct account of the different MT types, offering some detail of each type and elucidating the features that should characterise the MT engine that should be chosen for a particular translation assignment. MT quality assessment is then addressed, placing emphasis on the use of comparison for the purpose of evaluating the quality of different MT outputs. Three different types of evaluation of MT output quality will then be identified, namely: human evaluation, automatic evaluation and post-editing, offering specific detail in each section and showing how HTER metric is achieved. The method followed in the current research is then presented, which consists of a comparison between five different MT outputs based on post-editing grounded in HTER metric in Arabic–English legal translation to test which one has the highest output quality, followed by analysis of the results of the HTER metric. This is followed by data analysis carried out using 14 tables. The paper devotes a complete section to discussing the results of the data analysis, followed by some concluding remarks. The present paper argues that the use of HTER metric is a useful tool for the sake of undertaking post-editing procedures as it is a combination of both human evaluation as well as automatic evaluation. It is also advantageous as it takes account of both the use of lexicon and word order. However, HTER metric cannot be sufficiently depended on as one term substitution, which will be counted according to HTER metric as a single error, may render the whole sentence invalid, particularly in legal translation.

## 2 Types of MT systems

MT systems can be divided into two main systems: rule-based machine translation (RBMT) and corpus-based machine translation (CBMT) (Costa-Jussà et al., 2012). In the RB paradigm, expert linguists set out specific rules to explain the translation process, thus requiring a copious amount of input from the side of expert linguists (Dorr, 1994). Indeed, RBMT requires providing the programme with all the words specific to both the source and target languages needed alongside the specific rules used to structure well-constructed sentences in the languages concerned. The way in which the words of one language are linked to the words of the other language should then be specified, providing the programme with gradual instructions related to the way in which such details are made use of to produce translated text (Kenny, 2022). One disadvantage of RBMT is that it is costly to develop, requiring highly skilled and expert linguists to give the rules particular to each language pair (Goodfellow et al., 2016), in addition to the impossibility of anticipating all the knowledge demanded to operate such system as required (Kenny, 2022).

On the other hand, according to the CB paradigm, the knowledge extraction is automatically carried out through translation examples analysis based on a parallel corpus built by expert linguists. Upon the development of the appropriate techniques for a particular language pair, MT systems are then established to include new language pairs, making use of given training data (Costa-Jussà et al., 2012). This is the reason behind calling such paradigm data-driven MT where machine learning comes in. It relies on the premise that the programme would acquire its knowledge rather than being provided with the knowledge it needs. It does that through observing the way in which the problem, the machine seeks to resolve, had been resolved previously (Kenny, 2022).

Two other approaches can emanate from the CB paradigm or what is called data-driven MT, namely: statistical machine translation (SMT) and neural machine translation (NMT). SMT has two statistical models on the basis of the training data. The first model, which is called the translation model, is considered a bilingual model where words and phrases are presented in a table along with their translations as identified in the receptor language side of the training data, with each source-target language pair is given a probability score. Parallel examples in SMT, however, are employed for the sake of training a statistical translation model (Costa-Jussà et al., 2012; Kenny, 2022). The second model, which is said to be the language model, is a monolingual one or a combination of models of the receptor language. Both models are grounded in *n*-grams (Kenny, 2022). Based on the foregoing, SMT is contingent upon statistical criteria and a combination of translation and language models alongside the features that drive data (Costa-Jussà et al., 2012). In brief, SMT typifies translation knowledge in phrase tables and receptor language knowledge in independent *n*-gram models (Kenny, 2022). However, current MT systems seek to present a certain degree of analysis to the SMT (Costa-Jussà et al., 2012). It is noteworthy that there exist research works that employ both RBMT and SMT approaches (Matusov et al., 2008).

NMT, on the other hand, utilises examples that have already been presented in parallel corpora. Such MT system makes use of a bilingual corpus of parallel texts as its crucial knowledge foundation. It is indeed a translation by analogy and is regarded as an application of a reasoning approach of machine learning based on a particular case (Costa-Jussà et al., 2012). It is argued that NMT is able to learn metaphors and idiomatic expressions, adopting cultural equivalent in the receptor language rather than adhering to literalism (Marking, 2016). Such improvement in translation output made by NMT, which makes it more advanced than SMT may be due to the type of representations NMT generates and in the type of models it learns (Kenny, 2022; O'Brien, 2022). The models used by NMT systems are inspired by the human brain. NMT systems use artificial neural networks where thousands of independent units or artificial neurons are connected to other numerous artificial neurons (Kenny, 2022). It is indeed the activation states of a large number of connected neurons that can be comprehended as representing independent words and their link to other words (Forcada, 2017). As the current research is concerned with legal articles of the Saudi Commercial Law, it is worth pointing out that technology within legal sphere has unequivocally made a noticeable progress in the form of artificial intelligence, machine learning, deep learning and natural language processing worldwide. The legal informatics, as compared to technological advancements in other fields, such as health and medicine, has undeniably progressed despite the intricate nature of legal systems (Sharma et al., 2021). This with no doubt encourages reliance on MT systems in rendering legal materials from one legal system into another alongside assessing the output quality of such systems to ascertain as to whether or not such MT systems are valid for legal translation.

To choose the appropriate engine of a particular MT system, different features should characterise that very engine to achieve the intended goals. Amongst these are the suitability of the engine for the privacy requirements of the client, the buildability of the engine into the workflow concerned, the availability of the language pair required and the provision of the output that is easy to post-edit to meet the requirements of the client. The engine output quality hinges upon the special nature of the domain and text type, the trainability and usability of the engine as well as the pre-editing and post-editing effort that the translator is willing to exert (Rossi and Carré, 2022).

## 3 MT quality assessment

Translation quality assessment or what is called translation evaluation is substantial to translation (Munday, 2012; Alwazna, 2016b). Evaluation points generally to the specification of worth, merit or significance (Scriven, 2007; Alwazna, 2015). Translation quality is viewed as the quality of both the end-product and that of the transaction (Gouadec, 2010). In other words, quality in translation involves both the process as well as the product. Translation quality assessment also relies on the context in which the translation is accomplished alongside the user's needs and expectations (Rossi and Carré, 2022). To put it differently, translation quality is deemed a relative process (Grbić, 2008). In the field of translation studies, the concept of translation quality has been variably viewed and difficult to define; certain research studies ascribe changes in translation theories to the difference in scholars' views of translation quality, such as Drugan (2013) and House (2015).

Within the context of MT quality, Doherty (2017) claims that the result of the widespread use of translation technologies, particularly MT has brought about a plethora of pragmatic definitions pertinent to MT quality and measures. He goes on to point out that MT quality has been regarded as a means for achieving product, i.e. for the sake of improving MT systems, thus placing emphasis on a pragmatic approach that involves a mixture of both human and automatic evaluations. From the MT user's perspective, MT output quality is not easy to assess. It is claimed that MT quality is predominantly dependent on the system employed, the translation context along with the users' needs; all these are considered pivotal factors that need to be born in mind when assessing MT quality (Rossi and Carré, 2022). The pragmatic approach to MT quality assessment may imply adopting indicators that can be measured, such as user satisfaction ratings, increase in sale of a particular product based on machine translated instructions, increased productivity in post-editing and so on (Rossi and Carré, 2022).

It is noteworthy that if the translation evaluation is assigned to a human evaluator, the evaluation process will unquestionably involve subjectivity as evaluators may disagree on the quality level of a specific translation. Conversely, human translation evaluation can be objective when it is grounded in productivity measures (Rossi and Carré, 2022). Needless to say, human evaluation is deemed time-consuming and an in-depth resource process. Algorithms can be implemented instead to make an automatic evaluation, albeit irrelevant in certain situations that target specific applications (Rossi and Carré, 2022). The choice of a particular evaluation type should be founded on the nature of the translation project concerned and required needs.

One way to assess the MT quality is to compare different MT systems' outputs, as is the main theme of the present paper, to test their outputs in order to identify the best of them to be the translator's MT solution. Comparative analysis of different MT systems' outputs may be carried out to explore the errors made by each of them, classifying such errors into different categories, as will be addressed in the paper in question. This would assist the MT developers to further develop the systems and avoid such errors. There are various ways to compare outputs based on the question(s) that need to be answered. The scores adopted for such comparison may be founded on human evaluation, automatic evaluation and/or human post-editing effort (O'Brien, 2022; Rossi and Carré, 2022). Needless to say, the MT output that requires little post-editing is better and more advanced than that which demands a lot of post-editing where post-editing effort serves as a factor to assess MT quality in the case of accepting the logical assumption of using the machine translated texts for the purpose of dissemination (Kenny, 2022).

### 3.1 Human evaluation

Human evaluation points to the dependance on the human evaluator in the assessment of the MT system's output. Such evaluation is often carried out at a sentence level (Rossi and Carré, 2022), or even at a text level (Castilho, 2020). In human evaluation, segments are scored with the use of two different criteria, namely: adequacy and fluency. The former is assessed on an ordinal scale. It is a scale ranging from 1, which indicates that none of the meaning conveyed in the source segment is transmitted in the machine translated segment, to 4, which indicates that all the meaning given in the source segment is completely relayed in the machine translated segment (Rossi and Carré, 2022). On the other hand, the latter measures the degree to which the machine translated text follows the grammatical rules and norms of the receptor language (Castilho et al., 2018). Such criterion does not require assessing the machine translated text against the source text. The four-point ordinal scale, according to Castilho (2020), is also used in the assessment of fluency; 1 indicates that the machine translated segment does not follow the grammatical rules and norms of the target language, whilst 4 indicates that the machine translated segment is a native-like segment; it is formulated in accordance with the rules and norms of the target language.

A faster, yet more flexible human evaluation approach is to compare different MT systems' outputs by ranking each of them without justification. Such approach was used by Microsoft to elicit users' views on its different SMT and NMT outputs in 2017, as stated by Moorkens (2018). Other MT providers have relied on different interfaces to facilitate the human translation evaluation of MT outputs. For instance, Kantan AI provides a tool known as KantanLQR for the sake of language quality review, which enables users to determine the quality criteria that are most appropriate for their purposes and conducts a comparative analysis of four different MT outputs on the basis of the quality criteria specified. Such tools offer visualisation in the form of pie charts and bar charts of human evaluation scores for segments where evaluators are able to compute their overall scores for different MT systems' outputs (Rossi and Carré, 2022). Other tools, such as PET, are also available to assist in human evaluation of MT systems' outputs and are often employed by researchers (Aziz et al., 2012).

Errors made by each MT system will be classified into different categories of error typology. This is to diagnose the problems found in MT outputs, which would serve as a useful feedback to system developers (Rossi and Carré, 2022). Human translation may also contribute to the evaluation and development of MT output as well as the examination of the MT output problems. What is more, the majority of the current MT systems are dependent on human translation in learning the way in which they accomplish the translation task (Kenny, 2022). The categories particular to error typology drawn on in the present paper will be those suggested by Moorkens (2018) for the purpose of a practical in-class translation evaluation exercise. Such categories include word order errors (incorrect word order at phrase or word level), mistranslations (incorrectly translated word, wrong gender, number or case), omissions (words from the source text have been omitted from the target text) and additions (words not in the source text have been added in the target text).

### 3.2 Automatic evaluation

Automatic evaluation entails the use of automatic evaluation metric (AEM), which is doubtlessly faster and cheaper than human evaluation. For instance, in engine training, setting tests after each change enables the user to ascertain the improvement of the engine efficiency for the purpose specified. AEM also enables the user who is making use of a particular MT engine to receive multiple MT outputs for a single source text (Rossi and Carré, 2022).

Taken on board that advanced MT systems, which differ from other MT systems, are similar to human translations, several AEMs are grounded in the principle of similarity. Based on the foregoing, the evaluation tool is appropriate for both a human-generated gold standard or reference translation and the system output, which is called the candidate translation or hypothesis. A comparison is then made between the candidate and the reference translation followed by computing the similarity or dissimilarity. Having considered the variation and differences found in reference translations, certain evaluation tools may be fed various reference translations (Rossi and Carré, 2022). It is worthwhile that the exact computation of a particular AEM score may vary on the basis of the specific application detail peculiar to each metric. In other words, using different tools for the computation of the same AEM would yield different results. These different results are originated from specific factors, such as the way in which the tool copes with quotation marks, hyphens, etc., the way in which the tool defines tokens, its sensitive nature towards case and metric parametrisation specifics (Rossi and Carré, 2022). Four concepts are pivotal for the constitution of a more intricate AEM, namely: *n*-gram, precision, recall and F-measure. The concept of *n*-gram refers in translation to *n*-word sequences. Precision points to the ratio of correct words in the candidate translation, i.e. MT output, which also exist in the reference translation, i.e. human translation, to the overall number of words in the candidate translation. On the other hand, recall denotes the ratio of correct words in the candidate translation to the overall number of words in the reference translation. F-measure can be counted by multiplying the percentage of precision by the percentage of recall, divided by the total number of precision plus recall, multiplied by 2. Having considered the three metrics: precision, recall and F-measure, the higher score, the more advanced the MT output is. Nevertheless, such metrics are only used at the word level and do not take the word order accuracy into consideration (Rossi and Carré, 2022).

Translation error rate also known as translation edit rate (TER) counts for word order. It is founded on the word error rate (WER), which makes use of the Levenshtein distance, which computes the difference between word sequences in the candidate and reference translations, i.e. the editing steps that involve insertions, deletions and substitutions required to match the two sequences in question. WER normalises such distance through the length of the reference translation (Koehn, 2010).


$$\begin{array}{l} WER=\frac{no.\;of\;insertions\;+\;no.\;of\;deletions\;+\;no.\;of\;substitutions\;}{no.\;of\;words\;in\;the\;reference\;translation} \end{array}$$

When word sequences or clauses are shifted elsewhere in a particular sentence, each word shift counts as two errors; one is for deletion from its appropriate position in the sentence and one is for insertion in a different position. This may result in a poor MT output. TER minimises these two errors and makes it one by adding a shift operation, which denotes that moving any word sequence counts as one error (Rossi and Carré, 2022).


$$\begin{array}{l} TER=\;\frac{\begin{array}{c} no.\;of\;insertions\;+\;no.\;of\;deletions\\ +no.\;of\;substitutions\;+\;no.\;of\;shifts \end{array}}{no.\;of\;words\;in\;the\;reference\;translation} \end{array}$$

### 3.3 Post-editing

Post-editing refers generally to the task performed by the human evaluator to identify and fix the errors made by MT systems. It is considered a bilingual language-processing assignment, which is usually undertaken by expert translators (O'Brien, 2011, 2022; Nitzke and Hansen-Schirra, 2021). Contrary to what some translators believe that human translation is faster than post-editing, studies have proved that post-editing takes unquestionably less time than human translation (Guerberof Arenas, 2014). HTER, which will be employed in the current research, refers to the human evaluation through post-editing an MT output and counting the number of the editing steps required for the transformation of the MT output into the post-edited version (Snover et al., 2006). The more changes required for post-editing a particular MT output, the lower quality it has.


$$\begin{array}{l} HTER=\;\frac{\begin{array}{c} no.\;of\;insertions\;+\;no.\;of\;deletions\\ +no.\;of\;substitutions\;+\;no.\;of\;shifts \end{array}}{no.\;of\;words\;in\;the\;post-edited\;translation} \end{array}$$

Doherty (2017) claims that the combination of both automatic as well as human evaluation is deemed helpful, though the evaluator might encounter some variations. One important way to combine both measures is to exploit HTER, which provides a technical post-editing effort measure and a temporal measure, which informs of the time period taken to achieve post-editing (O'Brien, 2022). However, the present paper will only adopt technical post-editing measure as it focuses on assessing the quality of five MT outputs and identifying the highest MT system quality with regard to the rendition of Arabic legal articles into English.

Two different levels of post-editing can possibly be distinguished: light post-editing and full post-editing. The former points to the notion that only major errors made by MT output should be fixed as rapidly as possible. On the contrary, the latter means that all the errors made by MT output should be rectified, while such process should, of course, take a longer time when compared to the former (O'Brien, 2022). Full post-editing will be carried out in the present paper as the text that will be post-edited is legal which demands accuracy and precision (Alwazna, 2013a). The International Standards Organisation (ISO) has established a standard for post-editing, which is called “ISO 18857:2017.” According to this standard, light post-editing is defined as: “the process of post-editing to obtain a merely comprehensible text without any attempt to produce a product comparable to a product obtained by human translation” (ISO, 2017, p. 2). Conversely, full post-editing is defined according to the same standard as: “the process of post-editing to obtain a product comparable to a product obtained by human translation” (ISO, 2017, p. 2). It is worth pointing out that light post-editing may vary from one organisation to another depending on each organisation's requirements and what each organisation views it as major/essential error that needs to be fixed or otherwise. The objectives of post-editing, in accordance with the ISO 18857:2017 standard, lie chiefly in ensuring that the post-edited version is comprehensible, in line with both source and target language contents and compliant with the post-editing requirements specified by the translation service provider (ISO, 2017). Such objectives can be achieved through ascertaining that specific criteria are wholly fulfilled, such as consistency in the use of terminology, use of target language syntax and orthographic conventions, conformity with applicable standards, use of appropriate formatting, appropriateness for the purpose of the target language content and suitability for the target reader as well as compliance with the agreement concluded between the user and the translation service provider (ISO, 2017). Finally, there are certain characteristics suggested by De Almeida and O'Brien (2010) that should characterise the good post-editor; amongst these is that the post-editor should be able to recognise the MT issues that need to be post-edited and how they are best rectified. The post-editor should be sufficiently fast in performing such task so as to live up to the expectations specific to such activity. He/She should abide by the instructions given in order to reduce the preferential changes and the unnecessary ones.

## 4 Method

The present paper carries out an evaluation on five different MT outputs on the basis of post-editing procedures. In other words, each MT output is assessed against the same post-edited version, and the closest MT output to the post-edited version with regard to the use of the same lexicon and word order will achieve the lowest score. The lower the score of the MT output is, the higher quality it has. The MT systems involved in the study are considered amongst the most commonly used MT systems, particularly in the translation from and into Arabic. These are: Google Translate, hereafter (C1), PROMT.One, hereafter (C2), SYSTRAN Translate, hereafter (C3), Microsoft Bing, hereafter (C4), and Translate.com, hereafter (C5). The text used in the present study represents two legal articles: Article 17 and Article 108 taken from the Saudi Commercial law (Law of the Commercial Court) (1931). The choice for this particular sample has randomly been made. The metric used for evaluating the MT outputs in question is HTER, which is considered one of the most commonly used metrics in recent literature for the purpose of evaluating MT outputs with the use of post-editing procedures. It is a mixture of both human evaluation and automatic evaluation in the sense that it uses the metric of the latter, i.e. TER, while it is based on human post-editing.

Errors made by each MT system will be classified into different categories of error typology. The categories particular to error typology drawn on in the present paper will be those suggested by Moorkens (2018) for the purpose of a practical in-class translation evaluation exercise. Such categories include word order errors (incorrect word order at phrase or word level), mistranslations (incorrectly translated word, wrong gender, number or case), omissions (words from the source text have been omitted from the target text) and additions (words not in the source text have been added in the target text). Such categories are met by the Levenshtein distance with its operations or editing steps as proposed by Koehn (2010), which will be used in the present paper. Each operation or editing step suits a particular category. Such distance, which is used by WER, which serves as the basis of TER, computes the difference between word sequences in the candidate and reference translations. Likewise, such distance, in the case of using HTER, which will be adopted in the current research, computes the difference between word sequences in the candidate and post-edited version. The category of word order errors fits shift operation, whilst that of mistranslations is in line with substitutions. The category of omissions suits deletions, whereas that of additions goes hand in hand with insertions. The results of HTER metric evaluation will then be analysed to ascertain as to whether or not the MT outputs of high quality always produce acceptable Arabic–English legal translation.

The paper in question makes use of 14 tables; seven of which deal with the output quality of the five MT systems concerned in rendering Article 17 of the Saudi Commercial Law into English, whilst the other seven address the output quality of the same MT systems in translating Article 108 of the same law into English. Both Tables 1, 8 present the source texts of Article 17 and Article 108 respectively, the English translation of the MT systems concerned as well as the post-edited versions. Tables 2–6 show the operations required for the transformation of the five MT systems into the post-edited version respectively with regard to the rendition of Article 17 into English. Likewise, Tables 9–13 present the operations needed for the transformation of the same MT systems into the post-edited version respectively concerning the translation of Article 108 into English. Both Tables 7, 14 demonstrate HTER scores for each MT system with regard to the rendition of both Article 17 and Article 108 respectively. The equation specific to HTER metric is illustrated in Section 3.3 above.

**Table 1:** Source text of Article 17 of Saudi Commercial Law, the English translation of 5 machine translation systems and the post-edited version.

**ST**	**C1**	**C2**	**C3**	**C4**	**C5**	**PE**
كلالشركاتتقسمأرباحهاعلىالوجهالذيوقعالاتفاقعليهوبينالشركاء	All companies divide their profits according to the agreement signed between them and the partners	All companies divide their profits in the manner they signed the agreement and between partners	All companies divide their profits according to what has been agreed upon and among partners	All companies divide their profits in the manner agreed upon and between the partners	All companies divide their profits in the manner agreed upon and between the partners	All companies divide their profits in the manner agreed upon among partners

**Table 2:** Operations required for the transformation of candidate 1 into the post-edited version.

**Operation**	**Edited words**	**Number of edited steps**
Matches	All, companies, divide, their, profits, the, partners	7
Shifts		0
Substitutions	According\in, agreement\manner, signed\agreed, between\upon, them\among	5
Deletions	To, and, the	3
Insertions		0

**Table 3:** Operations required for the transformation of candidate 2 into the post-edited version.

**Operation**	**Edited words**	**Number of edited steps**
Matches	All, companies, divide, their, profits, in, the, manner, partners	9
Shifts		0
Substitutions	Signed\agreed, the\upon, between\among	3
Deletions	They, agreement, and	3
Insertions		0

**Table 4:** Operations required for the transformation of candidate 3 into the post-edited version.

**Operation**	**Edited words**	**Number of edited steps**
Matches	All, companies, divide, their, profits, agreed, upon, among, partners	9
Shifts		0
Substitutions	According\in, to\the, what\manner	3
Deletions	Has, been, and	3
Insertions		0

**Table 5:** Operations required for the transformation of candidate 4 into the post-edited version.

**Operation**	**Edited words**	**Number of edited steps**
Matches	All, companies, divide, their, profits, in, the, manner, agreed, upon, partners	11
Shifts		0
Substitutions	Between\among	1
Deletions	And, the	2
Insertions		0

**Table 6:** Operations required for the transformation of candidate 5 into the post-edited version.

**Operation**	**Edited words**	**Number of edited steps**
Matches	All, companies, divide, their, profits, in, the, manner, agreed, upon, partners	11
Shifts		0
Substitutions	Between\among	1
Deletions	And, the	2
Insertions		0

**Table 7:** Human translation edit rate scores for each candidate translation.

**Metric**	**C1**	**C2**	**C3**	**C4**	**C5**
HTER	67%	50%	50%	25%	25%

**Table 8:** Source text of Article 108 of Saudi Commercial Law, the English translation of 5 machine translation systems and the post-edited version.

**ST**	**C1**	**C2**	**C3**	**C4**	**C5**	**PE**
إعلانالإفلاسإماأنيكونبطلبمنالمفلسمباشرةأوبطلبمنأحدغرمائه	The declaration of bankruptcy is either at the request of the bankrupt directly or at the request of one of his creditors	The bankruptcy declaration is either at the request of the bankrupt directly or at the request of one of his fines	Declaring bankruptcy is either directly ordered by the bankrupt or at the behest of one of his suitors	Declaration of bankruptcy is either at the request of the bankrupt directly or at the request of one of his rivals	Declaration of bankruptcy is either at the request of the bankrupt directly or at the request of one of his rivals	The declaration of bankruptcy is made either at the request of the bankrupt directly or at the request of one of his creditors

**Table 9:** Operations required for the transformation of candidate 1 into the post-edited version.

**Operation**	**Edited words**	**Number of edited steps**
Matches	The, declaration, of, bankruptcy, is, either, at, the, request, of, the, bankrupt, directly, or, at, the, request, of, one, of, his, creditors	22
Shifts		0
Substitutions		0
Deletions		0
Insertions	Made	1

**Table 10:** Operations required for the transformation of candidate 2 into the post-edited version.

**Operation**	**Edited words**	**Number of edited steps**
Matches	The, bankruptcy, declaration, is, either, at, the, request, of, the, bankrupt, directly, or, at, the, request, of, one, of, his	20
Shifts		0
Substitutions	Bankruptcy\declaration, declaration\of, fines\creditors	3
Deletions		0
Insertions	Bankruptcy, made	2

**Table 11:** Operations required for the transformation of candidate 3 into the post-edited version.

**Operation**	**Edited words**	**Number of edited steps**
Matches	Bankruptcy, is, either, directly, the, bankrupt, or, at, the, of, one, of, his	13
Shifts		0
Substitutions	Declaring\the, by\at, behest\request, suitors\creditors	4
Deletions	Directly, ordered	2
Insertions	Declaration, of, made, request, of, the, directly	7

**Table 12:** Operations required for the transformation of candidate 4 into the post-edited version.

**Operation**	**Edited words**	**Number of edited steps**
Matches	Declaration, of, bankruptcy, is, either, at, the, request, of, the, bankrupt, directly, or, at, the, request, of, one, of, his	20
Shifts		0
Substitutions	Rivals\creditors	1
Deletions		0
Insertions	The, made	2

**Table 13:** Operations required for the transformation of candidate 5 into the post-edited version.

**Operation**	**Edited words**	**Number of edited steps**
Matches	Declaration, of, bankruptcy, is, either, at, the, request, of, the, bankrupt, directly, or, at, the, request, of, one, of, his	20
Shifts		0
Substitutions	Rivals\creditors	1
Deletions		0
Insertions	The, made	2

**Table 14:** Human translation edit rate scores for each candidate translation.

**Metric**	**C1**	**C2**	**C3**	**C4**	**C5**
HTER	4%	22%	57%	13%	13%

## 5 Discussion

As shown in Table 1, which demonstrates the ST of Article 17 of the Saudi Commercial Law, the outputs of C1, C2, C3, C4, C5, and the PE version, it is evident that both C4 and C5 have achieved the lowest score 25% in accordance with HTER metric, as presented in Table 7, which means that their output qualities are the highest of the five MT systems in translating this particular article into English with regard to matching them with the PE version in both the use of lexicon and word order. This is followed by C2 and C3, which have both achieved 50%, followed by C1, which has achieved 67%, as presented in Table 7. C4 and C5, which are surprisingly identical, have verbatim followed the PE version with the exception of two additions and one substitution. Such additions are typified by the two words; the connective and the definite article: “and, the” whose presence does not affect the intended legal meaning of the article concerned. Similarly, the use of the preposition: “between” by both C4 and C5 instead of the preposition: “among” employed by the PE version has no bearing on the appropriate legal meaning of the article in question. Given that the PE version is a literal rendition of the ST with the exception of the Arabic relative clause: “الذي وقع الاتفاق عليه,” which has been transferred into English with the use of the contact passive clause: “agreed upon,” C4 and C5 are considered to have literally adhered to the ST. Such adherence to literalism has been useful as the intended legal meaning of this article is the literal one. Moreover, literalism, i.e. faithfulness to the ST is unequivocally recommended in legal translation to preserve the letter of the law (Šarčević, 1997; Wolff, 2011; Alwazna, 2016a). Based on the foregoing, it seems evident that the translations produced by both C4 and C5 of the article concerned are acceptable.

Although C2 and C3 have achieved 50%, which means that their output qualities are lower than those of C4 and C5, the translations that they have produced are still acceptable. The deviation from literalism followed by the PE version appears in C2 in: “they signed the agreement,” which has been given as a translation of the Arabic relative clause: “الذي وقع الاتفاق عليه” where C2 has produced the subject pronoun: “they,” which refers to the partners, alongside the element of signing, which have both not been overtly stated in the ST. C3, on the other hand, has made use of the relative clause: “what has been agreed upon,” which seems closer to the ST, though differs from the PE version in not omitting the relative pronoun: “what” and the parts of the verb phrase of the clause: “has been.” Furthermore, C3 has employed the prepositional phrase: “according to” instead of the prepositional phrase: “in the manner,” which does not produce a totally different meaning, though the two phrases are not semantically identical. What is more, the connective: “and,” which is a literal translation of the Arabic connective: “و”, which has existed in both C2 and C3 should be dispensed with as it does not produce an idiomatic English legal text. The preposition: “between” in C2 should be replaced by the preposition: “among” for the sake of the idiomaticity of the text. Assessing both C2 and C3 against the PE version, it appears that C2 has added the third person plural pronoun: “they,” the noun: “agreement” and the connective: “and.” It has also used the verb: “signed,” the definite article: “the” and the preposition: “between” instead of the verb: “agreed,” the preposition: “upon” and the preposition: “among” respectively. C3, on the other hand, has added the part of the verb phrase of the clause: “has been” and the connective: “and.” It has also employed the prepositional phrase: “according to” and the relative pronoun: “what” in place of the prepositional phrase: “in the manner.” However, despite the aforementioned changes made by C2 and C3 and the differences between them and the PE version, their translations are still acceptable. This is due to the fact that neither C2 nor C3 has substituted a legal term with an inappropriate one, nor has any of them omitted a term that is deemed substantial to the collective legal meaning of the article in question. Therefore, although both C2 and C3 have achieved the same percentage 50% according to HTER metric, they differ in structure and are less idiomatic than the PE version, albeit acceptable in this particular legal context.

C1 has achieved the lowest output quality: 67%, as indicated in Table 7. It resembles C3 in employing the phrase: “according to” in place of “in the manner,” which has been used in the PE version. It is also akin to C2 in introducing the element of signing, which has not been clearly stated in both the ST and the PE version as: “the agreement signed between them,” which has been a rendition of the Arabic relative clause: “الذي وقع الاتفاق عليه”. It is noteworthy that in the C1 phrase, there has been a mention of an object pronoun: “them,” which again has not been indicated in the ST. Both the connective: “and” as well as the definite article: “the” should be removed and replaced by the preposition: “among.” Assessing C1 against the PE version, it seems evident that C1 has three additions, namely: the preposition: “to,” the connective: “and” and the definite article: “the.” Furthermore, C1 has employed the preposition: “according,” the noun: “agreement,” the verb: “signed,” the preposition: “between” and the pronoun: “them” in place of the preposition: “in,” the noun: “manner,” the verb: “agreed,” the preposition: “upon” and the preposition: “among” respectively. However, even though C1 has achieved 67% according to HTER metric, as indicated previously, the translation it has produced is still acceptable. The reason is the same as that for both C2 and C3, which lies in the fact that C1 has not substituted a legal term with an inappropriate one that would change the intended legal meaning of the article concerned, nor has it deleted a term that is pivotal for the collective legal meaning of the article under study, rather all the errors made thereby affect the idiomaticity and naturalness of the English legal text.

As indicated above and regardless of the evaluation based on HTER metric, all C1, C2, C3, C4, and C5 have generally managed to render the legal meaning of the article in question into English. However, there has been a varying degree concerning each MT output with regard to its translation quality. Taken on board that all the MTs involved in the current research are fluent and advanced as they have been successful in coping with Arabic legal translation, it is highly recommended that extra caution needs to be exercised to avoid making errors, particularly if the MT output is considered fluent. This is lent credence by Yamada (2019), who confirms that the more advanced the MT outputs are, the more challenging the process of post-editing at a professional standard is for student translators. Familiarity with the recurrent problems specific to MT outputs for a particular domain in a particular language pair would unequivocally scaffold translators to detect such problems and resolve them more effectively. Attention needs to be paid to small mistakes made by advanced MT, as presented above, and needs should be considered prior to deciding on the use of a specific MT system (Rossi and Carré, 2022).

As presented in Table 8, which demonstrates the ST of Article 108 of the Saudi Commercial Law, the outputs of C1, C2, C3, C4, C5, and the PE version, it seems clear that C1 has achieved the lowest score 4% according to HTER metric, as shown in Table 14, which means that its output quality is the highest of the five MT systems in translating this particular article into English with regard to matching it with the PE version in both the use of lexicon and word order. This is followed by both C4 and C5, which have both achieved 13%, followed by C2, which has achieved 22%, followed by C3, which has achieved 57%, as demonstrated in Table 14. C1 has evidently adhered to the PE version with the addition of one word made by the PE version, namely: “made” for the purpose of the text idiomaticity, though C1 has been structured correctly. Both C1 and the PE version are considered literal renditions of the ST, which look precise as the literal meaning of the article concerned is intended. This is the typical translation approach adopted by legal translators when both the form and substance can be conveyed (Šarčević, 2000; Alwazna, 2013b). Taken this on board, the translation produced by C1 of the article concerned is evidently acceptable.

Again, for the second article under study, both C4 and C5 turn out to be identical, achieving 13%. They only deviate from the PE version in omitting two words, namely: the definite article: “the” and the verb: “made” and placing the noun: “rivals” instead of the noun: “creditors.” Otherwise, both C4 and C5 stick to the PE version, which are all, of course, deemed literal renditions of the ST. However, although C4 and C5 have achieved 13%, which places them in the second rank after C1, which has achieved 4%, evidence suggests that the translations they have produced may not work, particularly in the legal domain. This, however, is owing to the fact that both C4 and C5 have failed to render the intended legal meaning of the legal financial term: “غرماء” precisely as “creditors,” which is the English legal term used for such legal purpose and as an equivalent term for the Arabic term: “غرماء”. The term: “غرماء” is the plural of the term: “غريم,” which means “الدائن,” literally “the creditor” (Almunjid, 2001, p. 1052). Instead, C4 and C5 have wrongly employed the term: “rivals,” which is not a legal financial term that serves the appropriate purpose. The difference between the term: “creditors” used by the PE version and the term: “rivals” employed by both C4 and C5 is clearly manifested in the meaning conveyed by each of them. The term: “Creditors” is the plural of the term: “creditor,” which refers to the “one to whom a debt is owed” (Oxford Dictionary of Law, 2002, p. 127). On the other hand, the term: “rivals” is the plural of the term: “rival,” which points to “a person, group, or organisation that you compete with in sport, business, a fight etc” (Longman Dictionary of Contemporary English, 2005, p. 1422). Obviously, there is an undeniable difference in meaning between the term: “creditors” used by the PE version and the term: “rivals” employed by both C4 and C5, in addition to the fact that the latter is not indeed a legal financial term. Such issue cannot be forgiven in legal translation, particularly in a particular state law, such as the Saudi Commercial Law. It is argued that a number of MT users often encounter problems in translating metaphorical expressions and abstract concepts (Rossi and Carré, 2022). Hence one error of substitutions made by a particular MT may render its translation invalid, particularly if the word substituted is a legal term that should be adhered to in such legal context.

C2, on the other hand, which has achieved 22%, deviates from the PE version in three elements, two of which do not affect the intended legal meaning, whilst the third impacts the conveyance of the appropriate legal meaning of the article under study. The first deviation appears in the change of the phrase: “the declaration of bankruptcy” as included in the PE version into the phrase: “the bankruptcy declaration.” Such difference in word order affects the HTER metric and its operations as in Table 10, though it does not impact the intended legal meaning of the article in question as the structure used in C2 is acceptable in the legal domain. The second deviation lies in the deletion of the word: “made,” which is present in the PE version, which again has no bearing on the intended legal meaning of the article concerned. Conversely, the use of the term: “fines,” which is the plural of the term: “fine,” which means “a sum of money that an offender is ordered to pay on conviction” (Oxford Dictionary of Law, 2002, p. 203) in place of the term: “creditors” is a major error made by C2 as the former is not a synonym of the latter, which should be adopted in this particular legal context. What is more, the term: “fines” does not convey the legal financial meaning intended by this article, nor does it even impart part of the meaning conveyed by the term: “creditors.” Hence the translation produced by C2 has not managed to transfer the intended legal meaning of the article under study as a result of the inappropriate use of terms.

C3, which has the lowest output quality as it has achieved 57%, has deviated from the PE version in four different factors; three of which are insignificant as they have no influence on the intended legal meaning of the article concerned, whilst the fourth represents a substantial change in the intended legal meaning of the article in question. The first deviation of C3 is typified by the phrase: “declaring bankruptcy,” which reads in the PE version as: “the declaration of bankruptcy.” Such change may affect the formality of the phrase employed by C3, albeit with no bearing on the intended legal meaning of the article under study. The second deviation of C3 from the PE version resides in the clause: “is either directly ordered by the bankrupt,” while its counterpart in the PE version reads as: “is made either at the request of the bankrupt directly.” It is noted that C3 here has not restricted itself to literalism, rather it has enjoyed a leeway in rendering the intended legal meaning of this part of the article and has ipso facto managed to perform this task successfully. This supports the claim made by Marking (2016), who argues over the merit that NMT is able to avoid literalism, rephrasing the sentence in question based on its meaning. It is claimed that amongst the translation techniques used for the rendition of legal terms is paraphrase, or what is known as descriptive paraphrases (Arntz, 1993; Alwazna, 2019). The third deviation rests upon the substitution made by C3 where it has adopted the term: “behest” in place of the term: “request,” which has been utilised by the PE version. However, since both the terms designate the same concept and are both used in legal discourse, such substitution shall have no effect on the intended legal meaning of the article concerned. Nonetheless, the second substitution made by C3, which stands for the fourth deviation from the PE version is considered to be significant as C3 has made use of the term: “suitors” instead of the term: “creditors,” which has been adopted by the PE version. The term: “suitors” is the plural of the term: “suitor,” which is the active participle of the term: “suit,” which denotes “a problem or complaint that a person or company brings to a court of law to be settled” (Longman Dictionary of Contemporary English, 2005, p. 1661). Having examined the meaning of both terms: “suitors” and “creditors,” it seems evident that there is a clear difference in meaning between them, in addition to the fact that the term: “suitors” is not a legal financial term that is often used in commercial law. Hence C3 has made a major error, using the term: “suitors” in place of the term: “creditors,” which has rendered the intended legal meaning of the article in question void. In legal translation, translators should be amply sensitive to the terminology employed and have sufficient appreciation of accuracy in the use of appropriate structure of legal discourse (Smith, 1995; Alwazna, 2017). Likewise, MT developers should have the same sensitivity when dealing with implementable texts, such as legal texts.

Although the percentages in accordance with HTER metric achieved by C1, C2, C3, C4, and C5 in translating Article 108 of the Saudi Commercial Law into English is obviously lower than those achieved by the same MT systems in rendering Article 17 of the same law into English, which means that the quality of the said MT systems is higher in translating the former than in translating the latter, the translations of Article 108 produced by all the aforementioned MT systems are invalid except for that produced by C1. This is due to the fact that such MT outputs substitute a legal financial term with an inappropriate one, which is counted as only one error, thus rendering the collective legal meaning of the article concerned void.

## 6 Concluding remarks

This small-scale research has been carried out for the sake of quality assessment of five different MT outputs with regard to Arabic–English legal translation through post-editing. Two articles of the Saudi Commercial Law, namely: Article 17 and Article 108 have randomly been chosen to be translated by five MT systems through post-editing based on HTER metric. Such metric has proved fruitful as it combines both automatic evaluation as well as human evaluation in the sense that it employs the metric of the former, namely: TER metric, whilst it is accomplished through human post-editing. HTER metric is also useful as it is able to evaluate the whole text, working on a sentence level as it takes account of both the use of lexicon along with word order. Also, WER is often higher in TER metric rather than in HTER metric as the former assesses the MT version against a human translation version that was translated before the MT version, whilst the latter assesses the MT version against a post-edited translation version that has been achieved in view of the errors made by the MT version.

By contrast, HTER metric cannot be depended on individually, specially in Arabic–English legal translation as a particular MT system may have a high output quality according to such metric as it has only a single error, however, such error, particularly the term-substitution error, may affect the collective intended legal meaning of the text and may ipso facto render it completely invalid. On the other hand, the MT system may have a lower output quality according to HTER metric due to multiple errors. However, such errors do not impact the collective intended legal meaning of the text in question, hence the translation still remains acceptable, albeit unidiomatic. This is particularly true in the rendition of both Article 17 and Article 108 of the Saudi Commercial Law into English where all the five MT systems have translated the first article quite acceptably while having generally low output quality according to HTER metric, whereas the same systems have failed with the exception of C1 to translate the second article appropriately though they generally score higher output quality according to the same metric. Therefore, it is recommended that HTER metric is further developed in the sense that the editing steps are not identically counted and that the operation of substitutions counts more than other operations. This is due to the fact that the operation of substitutions may end up replacing an important legal term with a false one that does not designate the same legal concept as that conveyed by the substituted one. This has negative implications for the MT translation and can clearly render the whole text void if it is a legal text whose translation purpose is for application and implementation.

This paper is limited to comparing five MT outputs in rendering certain Arabic legal articles taken from the Saudi Commercial Law into English for output quality assessment through post-editing based on HTER metric. It has revealed certain merits and demerits for such metric in MT output evaluation. Other research is recommended for quality assessment of the same MTs outputs, for the same language pair and within the same domain, using other metrics, such as TER, BLEU and ChrF3. Further research is required to assess the same MTs outputs quality using other language pairs, employing various metrics and testing texts within diverse disciplines. Endeavours need also to be made to assess the quality of other MTs outputs, using the same pair, the same metric and the same domain to compare the results of the current research to those of the other research.

## Data availability statement

The original contributions presented in the study are included in the article/supplementary material, further inquiries can be directed to the corresponding author.

## Author contributions

RA: Writing—original draft.
